# The proximal and distal effects of mortality salience on COVID‐19‐related health perceptions and intentions

**DOI:** 10.1111/jasp.12903

**Published:** 2022-07-05

**Authors:** Samuel Fairlamb, Marco Cinnirella, Inbal Iahr

**Affiliations:** ^1^ Psychology Department, Royal Holloway University of London Egham UK

## Abstract

Health preventative measures are important in reducing transmission of COVID‐19, yet death‐related thoughts might hinder preventative action. Using two online samples (*N* = 948), we examined how mortality salience (MS) may produce health‐related proximal and distal defenses relevant to COVID‐19, examining health optimism and appearance self‐worth as moderators. MS decreased perceived vulnerability as a proximal defense for those with high health optimism (Study 1), while those with low health optimism decreased perceived response efficacy of face masks and intention to wear a protective face mask (Study 2). Additionally, those with high appearance self‐worth displayed increased intention to wear an aesthetically appealing face mask as a distal defense to MS (Study 2). Our findings demonstrate the importance of considering how mortality concerns may channel health‐defeating and health‐promoting behaviors in respect to COVID‐19 and provide insight into how to produce sustained engagement in health preventative action to combat the pandemic.

## 1 INTRODUCTION

In response to the COVID‐19 pandemic, citizens worldwide have been asked to engage in health preventative behaviors to help save lives. Although several models exist that illuminate on the antecedents of behavioral health intentions (e.g., Rogers, 1983; Witte, 1992), because the disease is associated with death, the Terror Management Health Model (TMHM: Goldenberg & Arndt, 2008) is uniquely posed to understand how mortality concerns may facilitate, or cause barriers, to health behavioural intentions. We present two studies that examine how the awareness of death may influence *proximal* and *distal* reactions relevant to COVID‐19 health behaviors.

## 2 TERROR MANAGEMENT HEALTH MODEL

The TMHM is an extension of the social‐psychological theory known as terror management theory (TMT; Greenberg et al., 1986), which stipulates that humans are oriented towards survival yet are acutely aware of the fact that someday they will die. This juxtaposition provides the potential for existential anxiety, which humans manage through a combination of *proximal* and *distal* defenses (Pyszczynski et al., 1999). Proximal defenses are initiated by conscious death thoughts and focus on attempting to push death thoughts outside of conscious awareness. Distal defenses are initiated by nonconscious accessibility of death thoughts and have no logical connection to death but attempt to manage the problem on a symbolic level by making one feel like they are valuable contributors to a meaningful universe (i.e., self‐worth).

The majority of studies examining TMT have deployed the mortality salience (MS) hypothesis (Burke et al., 2010). This hypothesis states that if psychological structures (i.e., self‐worth) manage death‐related concerns, then reminders of death should increase the need for those psychological structures. Hundreds of experiments have supported the various ways in which different individuals, from different cultures, go about managing death‐related concerns (Pyszczynski et al., 2015). Importantly, evidence also suggests that MS leads to immediate and active suppression of death‐related thoughts (Arndt et al., 1997), but after a brief (5–10 min) delay when death thoughts are no longer consciously accessible people engage in distal defenses (Burke et al., 2010). Taken together, this study gives credence for the two different defensive tactics to cope with the awareness of death.

The TMHM, therefore, applies TMT's ideas of how people manage the problem of death to the area of health (Goldenberg & Arndt, 2008). The model stipulates that when people are confronted with their mortality due to health threats, they first engage in proximal defenses that are threat‐focused attempts to push death thoughts outside of conscious awareness. However, the model further stipulates that when death thoughts are no longer conscious, people engage in distal defenses that aim to affirm a sense of cultural value and significance. As these defenses are not driven by health concerns but by self‐worth, they can produce outcomes that are health‐facilitating or health‐defeating depending on one's contingencies of self‐worth.

Recent work has speculated on the role that terror management processes may play in respect to health behaviors within the COVID‐19 pandemic (Courtney et al., 2020; Pyszczynski et al., 2020). However, research directly supporting these claims thus far is scarce (although see Courtney et al., 2021). The present research addresses this gap by examining how proximal and distal defenses may impact COVID‐19‐related health decisions. As terror management defenses are contingent on a host of health and self‐related variables, we examine health optimism (Aspinwall & Brunhart, 1996) and appearance‐contingent self‐worth (Crocker et al., 2003) as moderators to these defenses. Finally, we utilise insights from expectancy‐value models of health behaviors (e.g., Rogers, 1983; Witte, 1992) to help illuminate how people appraise health decisions in the face of MS.

### 2.1 Proximal defenses

According to the TMHM, health threats such as COVID‐19 can increase conscious thoughts about mortality that can initiate proximal defenses to manage said health threat. Depending on particular individual differences, these defenses can produce outcomes that are health facilitating or defeating (Arndt & Goldenberg, 2017). For example, proximal defenses can increase engagement with health behaviors (e.g., exercise, Arndt et al., 2003) or conversely deny one's vulnerability to these health threats that can increase avoidance of health behaviors (e.g., cancer screenings; Arndt et al., 2006).

Past research has examined the role of health optimism, which is the tendency to be optimistic about one's health and outcomes of health risks assessments, in relation to proximal health reactions to MS (Arndt et al., 2006; Cooper et al., 2010). These studies demonstrate how high health optimism (in comparison to low health optimism) may motivate increased engagement with health behaviors (e.g., cancer screenings) after MS because a sense of optimism may help people maintain confidence that they can effectively manage the threat. Indeed, optimism is associated with positive illusions about one's ability to control their own risk (e.g., McKenna, 1993; Weinstein, 1980), including the effectiveness of behaviors that can reduce one's risk to health threats (e.g., response efficacy; Taylor et al., 1992; Weinstein & Lyon, 1999). Optimists may therefore feel confident that threats are within their control thus increasing engagement in health preventative action. Stated differently, those who lack a sense of optimism may feel that certain behaviors are ineffective in controlling the threat. The relationship between optimism and perceived control may be one reason that optimism is linked with increased engagement in problem‐focused, rather than avoidance‐based, strategies (e.g., Aspinwall & Taylor, 1992; Scheier & Carver, 1993).

However, health optimism has the possibility to channel health‐defeating, as well as health‐promoting behaviors (Arndt et al., 2006). This is because optimism, under some circumstances, can lead to unrealistic biases (Tennen & Affleck, 1987; Weinstein, 1982), and decrease people's perceived vulnerability to health risks (e.g., Facione, 2002; Taylor et al., 1992). This could have the potential to decrease their willingness to engage in health preventative actions, including COVID‐19 health behaviors (e.g., Graffigna et al., 2020). Indeed, evidence suggests that optimists tend to underestimate their risk (e.g., being infected, having severe complications) to the COVID‐19 disease (Monzani et al., 2021).

Taken together, health optimism should increase the sense that one can effectively cope with health threats, but concomitantly decrease their sense that they are vulnerable. While this tendency might be present in control conditions, it is likely to be particularly pronounced under conditions of MS because conscious death thoughts should increase reliance on typical coping strategies that low and high‐health optimists use to manage threatening thoughts.

In line with expectancy‐value models of health behaviors (e.g., Rogers, 1983; Witte, 1992), whether these changes produce health defeating or facilitating outcomes is dependent on a balance between one's coping (i.e., response efficacy, self‐efficacy) and threat (i.e., perceived vulnerability and severity) appraisal. When threat appraisals are high and coping appraisals are low, this can cause counter‐intuitive outcomes of increasing engagement with maladaptive behaviors (Rogers & Prentice‐Dunn, 1997). In the context of the pandemic, this might result in low engagement in health preventative behaviors (e.g., handwashing, social distancing) that reflects a fatalistic attitude (Jimenez et al., 2020). Such outcomes are likely to be realised in the case of those with low levels of health optimism.

In contrast, while those with high optimism may have a lowered sense of threat appraisal, their increased sense of coping appraisal may help them feel more confident and capable of protecting themselves from health risks (even if such risks are diminished).^1^ This should increase engagement in health preventative action. From a TMHM perspective, the idea that certain behaviors are able to effectively manage health concerns should make them good actions to help manage thoughts about one's own mortality. Indeed, one can draw comparisons to research on fear appeals which suggests that they are more effective in producing behavioral intentions when they are accompanied by efficacy statements (e.g., Tannenbaum et al., 2015).

### 2.2 Distal defenses

While proximal defenses are engaged to combat conscious death thoughts, when successfully removed from conscious awareness, people engage in distal defenses to manage concerns about their mortality (Arndt & Goldenberg, 2017). Like with proximal defenses, distal defenses can produce health facilitating or health defeating outcomes, though the precise outcome depends on self‐related variables. Studies have demonstrated how different contingencies of self‐worth can influence distal responses to MS in a health domain. For example, immediately following MS, people show an increased intention to exercise as a proximal defense, but after a delay only those with fitness‐based contingencies of self‐worth show an increased intention to exercise (Arndt et al., 2003). Similarly, those who view smoking as a source of self‐worth show increased positive attitudes to smoking when exposed to death‐related cigarette warnings (Hansen et al., 2010).

Of particular interest in this study is how appearance‐based contingencies of self‐worth (Crocker et al., 2003) may shape distal responses to MS. As these individuals view their self‐worth as contingent on their appearance, this can drive health‐defeating outcomes in response to MS. For example, prior studies have shown that those with high appearance‐based contingencies showed less interest in purchasing highly protective sunscreen (Routledge et al., 2004) and more interest in tanning products (Arndt et al., 2009) as a distal response to MS.

In relation to the COVID‐19 pandemic, appearance‐based contingencies may shape reactions to face mask use. Face masks represent one way in which the transmission of the disease can be curtailed, thus initially serving as a proximal defense. However, it can also progress to being a distal defense when it becomes imbued with cultural significance (Courtney et al., 2020). More generally, in comparison to other pandemic‐related health behaviors (e.g., social distancing), face masks provide an obvious way for people to assert their cultural significance. For example, face masks are now available from various designer labels, and come in various colors, designs, patterns with symbolic logos and brandings. Indeed, face mask use is not always driven by health concerns, and in line with our assertions, how people feel about their appearance has been identified as an important issue in the wearing of face masks (e.g., Howard, 2020). Although the aesthetic qualities of a face mask would do little to protect oneself from a potentially lethal disease, the TMHM is well equipped to explain why some people, particularly those with high appearance contingencies, may base their choices to wear a face mask on such reasons.

## 3 STUDY 1

In sum, the present research sought to examine how MS may drive proximal and distal reactions that have relevance to COVID‐19 health outcomes. Both studies were conducted through the period of March–June 2021. In study 1, we examined our first prediction that MS would affect levels of perceived vulnerability to COVID‐19 as a proximal defense, and that this would be moderated by levels of health optimism. We predicted that immediately following MS, those with low health optimism would show increased vulnerability to COVID‐19, while those with high health optimism would show a decreased sense of vulnerability.

To examine this, participants completed a measure of health optimism before answering a traditional MS manipulation (vs. physical pain). Participants then answered questions about their vulnerability to COVID‐19 as well as the severity of the disease. While we limited our hypothesis to the vulnerability measure, we included severity for exploratory purposes as one could potentially envisage that people can minimise the sense of threat of the disease by dismissing its severity. We manipulated whether participants answered these questions immediately after MS or after a delay.

## 4 STUDY 1 METHOD

### 4.1 Participants

We collected English‐speaking participants via online forums and Amazon MTurk.^2^ In total, 663 participants took part, but only 583 completed it. We excluded 139 participants for the following reasons: (i) 82 participants either did not respond to the MS manipulation, or gave a nonsensical, or copy and pasted, answer from the internet; (ii) 19 failed one or more attention checks in the survey; (iii) 8 indicated they had taken part in a MS experiment before and/or indicated they knew the aim of the experiment; (iv) 3 suggested we should not use their data as it was unreliable; (v) 27 completed the delay materials under 2 min, which reduced confidence in the quality of their data (less than 2 s per item) and whether we were capturing proximal and distal defenses.

This left a final sample of 444 participants (*M*_age_ = 39.3, *SD*_age_ = 14.5) with 153 males, 283 females, and 8 who identified as nonbinary. Post hoc sensitivity analyses suggested we had 95% power to detect a small three‐way interaction effect (*f* ^2^ = 0.03). Participants were randomly allocated to one of four conditions: MS/Immediate (*n* = 115), MS/Delay (*n* = 108), control/Immediate (*n* = 116), control/Delay (*n* = 105). The study went through the university's standard ethical approval process.

### 4.2 Materials and procedure

Participants were informed the study concerned personality and health beliefs. They first completed a measure of health optimism, and then were subsequently primed for thoughts of death (vs. control). To measure proximal and distal defenses, participants either completed the dependent measures immediately or after a delay. Finally, participants answered some basic demographic information, questions relating to COVID‐19, and were probed about the study aims and their participation.^3^

#### 4.2.1 Health optimism

We measured health optimism (e.g., *I am healthier than most people I know*) in 8 items used in past research (Aspinwall & Brunhart, 1996). Participants responded to these items on 7‐point scales (1 = strongly disagree, 7 = strongly agree). We included an additional 3 filler items form the original scale to mask the intention of the scale. We computed a mean after reversing scoring the appropriate items (*α* = .86).

#### 4.2.2 Mortality salience

To manipulate MS, participants answered two open‐ended questions about their own death (e.g., Burke et al., 2010). Participants in the control condition answered parallel questions about experiencing physical pain.

#### 4.2.3 Delay/distraction

To ensure thoughts of death had left focal attention, participants in the delay condition first answered 30 items that measured types of affect (fear, anxiety, anger, sadness, happiness) used in past research (Lambert et al., 2014). They also completed a short‐form morningness questionnaire (Smith et al., 1989) and a 20‐item Big Five personality measure (Donnellan et al., 2006) before the dependent measures. Participants in the immediate condition completed these materials after the dependent measures.

#### 4.2.4 Perceived vulnerability and severity

We measured perceived vulnerability (e.g., “I would become very ill if I contracted the coronavirus”) and severity (e.g., “The coronavirus is a serious illness”) of COVID‐19 in three items each. Participants completed these items on 9‐point Likert scales (1 = strongly disagree; 9 = strongly agree). We computed mean scores for perceived vulnerability (*α* = .87) and severity (*α* = .82).^4^

## 5 STUDY 1 RESULTS

To test for the effects of MS and the delay at high and low levels of health optimism, we utilised Model 3 in PROCESS (v3.4; Hayes, 2018). MS (−1 Pain, +1 MS) was inserted as the independent variable, with the delay (−1 Immediate, +1 Delay) and health optimism were inserted as moderating variables. Health optimism was standardised. We regressed these variables onto our dependent variables perceived vulnerability and severity.^5^

### 5.1 Perceived vulnerability

**Figure 1:**
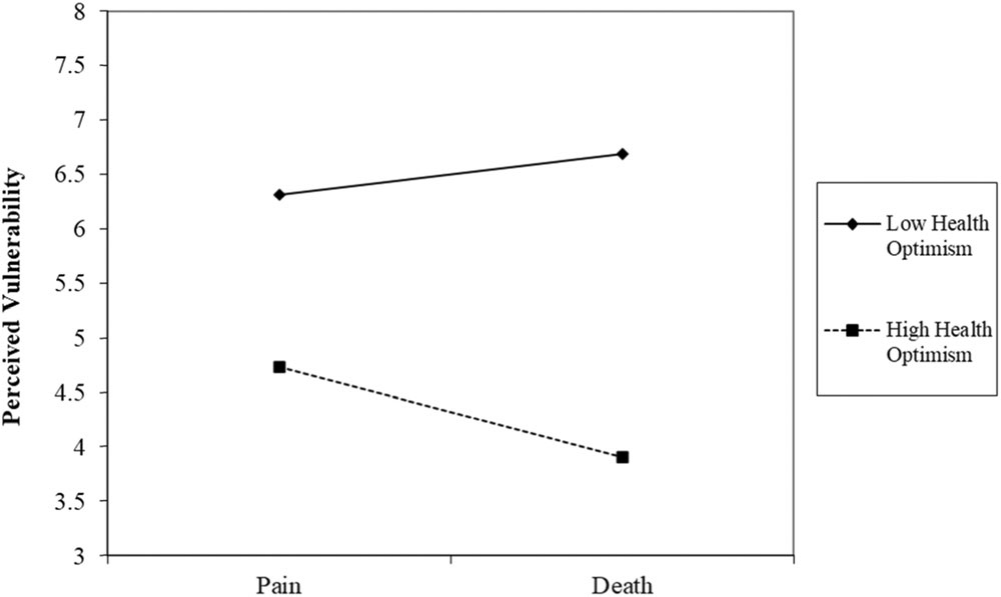
MS X Health Optimism on levels of perceived vulnerability in the immediate condition plotted at ±1 *SD* levels of health optimism

There was a main effect of health optimism decreasing perceived vulnerability, *β* = −1.03, *t* (436) = 11.39, *p* < .001, 95% confidence interval [CI] [−1.21, −0.85]. The only other effect that was significant was the predicted three‐way interaction, *β* = .19, *t* (436) = 2.14, *p* = .033, 95% CI [0.02, 0.37]. Examining the MS X Health Optimism interaction at each level of the delay showed that the interaction was significant in the immediate condition, *β* = −.30, *p* = .020, but not in the delay condition, *β* = .09, *p* = .494. Figure 1 displays this two‐way interaction in the immediate condition.

Simple slopes analyses plotted at high (+1 *SD*), and low (−1 *SD*) levels of health optimism showed that at high levels of health optimism, MS decreased levels of perceived vulnerability, *β* = −.42, *t* (436) = 2.42, *p* = .016, 95% CI [−0.75, −0.08]. MS increased levels of perceived vulnerability at low levels of health optimism albeit the effect was not statistically significant, *β* = .19, *t* (436) =0.99, *p* = .321, 95% CI [−0.58, 0.10]. Looking at the interaction differently by examining the effect of the delay showed that those with low health optimism in the MS group showed increased perceived vulnerability when measured immediately, *β* = −.38, *t* (436) = 2.12, *p* = .035, 95% CI [−0.74, −0.03]. There was no effect of the delay in those with high health optimism in the MS group and no other effects were statistically significant, *p*'s > .15.

### 5.2 Perceived severity

There was a main effect of health optimism decreasing perceived severity, *β* = −.47, *t* (436) = 6.34, *p* < .001, 95% CI [−0.62, −0.33]. There was also an effect of the delay with the immediate condition increasing perceived severity of the disease, *β* = −.15, *t* (436) = 2.07, *p* = .039, 95% CI [−0.30, −0.01]. No other effects were significant, *p*'s > .15.

### 5.3 Affect

We also examined whether our manipulations influenced levels of affect (fear, anxiety, happiness, sadness, and anger). We also considered the potential role of health optimism in these effects. There were no effects for anxiety, happiness, sadness and anger (*p*'s > 0.15). For fear, there was a two‐way interaction effect between MS X Delay, *β* = −.06, *t* (434) = 2.01, *p* = .046, 95% CI [0.00, 0.12].^6^ This was qualified by a marginal three‐way interaction, *β* = −.05, *t* (434) = 1.75, *p* = .081, 95% CI [−0.12, 0.01].

Examining the two‐way interaction between MS X Delay for those with low and high health optimism suggested that the interaction was significant in those with low health optimism, *β* = .12, *p* = .008, but not in high health optimism, *β* = .01, *p* = .859. Simple slopes analyses suggested that immediately after MS those with low health optimism showed increased fear albeit this was marginal, *β* = .08, *t* (434) = 1.73, *p* = .084, 95% CI [−0.01, 0.22]. After a delay, MS decreased fear for those with low health optimism, *β* = −.13, *t* (434) = 2.03, *p* = .043, 95% CI [−0.26, −0.00]. Looking at this interaction differently, there was only an effect of a delay in the MS group among those with low health optimism, *β* = .16, *t* (434) = 2.56, *p* = .011, 95% CI [0.04, 0.28].

## 6 STUDY 1 DISCUSSION

MS increased the tendency to deny one's perceived vulnerability to the COVID‐19 disease as a proximal defense. However, such an effect was limited to those high in health optimism. In contrast, those with low health optimism tended to increase their perceived vulnerability. However, we found this latter effect to only be significant when examining the effect of the delay in the MS group so some caution when interpreting this finding should be advised. No reliable effects were found for the effect of MS on perceived severity. We believe this makes sense as the TMHM speaks more to the role of how death awareness may motivate attempts to deny one's perceived vulnerability to health threats, rather than the severity of them.

Interestingly our analyses also suggested that MS increased levels of fear immediately, and decreased levels of fear after a delay for those with low health optimism. This could be consistent with our finding that those with low health optimism feel more vulnerable in comparison to those with high health optimism immediately after MS, thus exhibiting increased fear. It is not entirely clear why those with low health optimism would then exhibit decreased fear after a delay. One possibility is that because low optimism is associated with emotional avoidance and distancing (Nes & Segerstrom, 2006), the immediate increase in fear onset by MS leads to efforts to suppress them. Consistent with this, prior research has suggested that increasing perceived vulnerability to breast cancer increases suppression efforts after MS (Arndt et al., 2007). However, given that the findings concerning fear were marginal at best, and we did not hold any a priori predictions regarding affect, we would not wish to overinterpret these effects until validated in a new sample.

## 7 STUDY 2

To extend our findings, in Study 2, we focused on a specific COVID‐19 health behavior: face mask use. We focused on face masks because there is public debate about their perceived effectiveness (Kasting et al., 2020; Rieger, 2020), but moreover, they are also potentially a way for people to assert their cultural identity or self‐worth through a health preventative behavior (Courtney et al., 2020).

We had two primary aims. First, we wanted to further examine how health optimism shapes reactions to health issues by considering the role of response efficacy. Although Study 1 showed that high health optimism increases a tendency to deny vulnerability to COVID‐19, because optimists tend to feel that threats are within their control, MS should increase a sense that masks are effective ways of managing COVID‐19. Therefore, we anticipated that high health optimists would increase perceived response efficacy, while those with low health optimism should have decreased perceived response efficacy as they see health threats as less controllable. In turn, this should influence their decision to wear a face mask based on their clinical effectiveness.

Second, we also wanted to examine distal responses to MS by examining choices behind face mask use. In particular, we focused on whether people might find face masks that are aesthetically attractive as a factor in their decisions to wear one. As people vary regarding what is a relevant source of self‐worth, we expected this effect to only occur for those who had high levels of appearance contingent self‐worth. Thus, in general, we expected that proximal defenses would drive decisions surrounding the clinical effectiveness of face masks in their ability to reduce risk of infection. In comparison, we expected that distal defenses would drive concerns over self‐worth particularly regarding concerns over appearance.

Finally, we also had an ancillary aim due to the unanticipated effects pertaining to fear in Study 1. Thus, we sought to see if these would be replicated in a new sample using a different measure of affect.

## 8 STUDY 2 METHOD

### 8.1 Participants

We collected English‐speaking participants via online forums and social media. In total, 753 participants took part, but only 532 completed it. We excluded 28 participants using the same criteria from Study 1. This left a final sample of 504 participants (*M*_age_ = 25.7, *SD*_age_ = 8.5) with 171 males, 309 females, and 23 who identified as nonbinary (1 participant did not provide this information). Post‐hoc sensitivity analyses suggested we had 95% power to detect a small three‐way interaction effect (*f* ^2^ = 0.025). Participants were randomly allocated to one of four conditions: MS/Immediate (*n* = 121), MS/Delay (*n* = 124), control/Immediate (*n* = 127), control/Delay (*n* = 132). The study was ethically approved by the university.

### 8.2 Materials and procedure

Participants were informed the study concerned personality and health decisions. Participants first completed an appearance contingent self‐worth and health optimism measure before being randomly assigned to answer a death anxiety scale (vs. control). Participants then completed the key measures concerning the response efficacy of face masks, and their preferences for wearing a protective and aesthetic looking face mask either immediately or after a delay. Participants at the end of the study provided some basic demographic information, were probed for suspicion about the study, and answered whether they felt the data provided was reliable.

#### 8.2.1 Appearance contingent self‐worth

We measured appearance contingency in five items (Crocker et al., 2003) on a 7‐point scale (1 = *strongly disagree*, 7 = *strongly agree*). We created a mean score with higher scores reflecting increase contingent self‐worth (*α* = .83). We also measured other contingencies in this section to obscure our interest on appearance self‐worth.

#### 8.2.2 Health optimism

Participants completed a health optimism scale similar to Study 1 which consisted 13 items (*α* = .72), as well as three filler items to mask the aim of the measure.

#### 8.2.3 Mortality salience

To manipulate thoughts of death, participants in the MS condition answered 8 items from a death anxiety scale (e.g., *I am not at all afraid to die*; Templer, 1970). Control participants answered 8 items about general life statements (e.g., *Love is more important than money*).

#### 8.2.4 Delay materials

The delay consisted of the 60‐item Positive and Negative Affect Schedule—Expanded Form (Watson & Clark, 1999), which measures various types of affect including fear. Participants also completed the 20‐item Big Five personality inventory (Donnellan et al., 2006). Participants in the delay condition completed these items before the dependent measures, while participants in the immediate condition completed these items after the dependent measures.

#### 8.2.5 Face mask preference

We measured preference for types of face mask with eight items, four that related to whether a mask was clinically effective in protecting oneself (e.g., “I would choose a face mask which is recommended by doctors to keep me safe”) and four that related to whether the mask was aesthetically pleasing (e.g., “I would choose a face mask which makes me look good”). Participants answered on 9‐point scales (1 = *strongly disagree*, 9 = *strongly agree*). A principal components analysis suggested these were separate factors, so we computed mean scores for protection (*α* = .91) and aesthetic (*α* = .82) preferences.

#### 8.2.6 Face mask response efficacy

We measured the response efficacy of face mask usage in three items (*α* = .94; e.g., “I believe wearing a face mask is important in reducing my chance of infection”).^7^ Participants answered these items on 9‐point scales (1 = *strongly disagree*, 9 = *strongly agree*).

## 9 STUDY 2 RESULTS

### 9.1 Health optimism

As with Study 1, we utilised PROCESS (Hayes, 2018) to test for a three‐way interaction between MS (−1 Control, +1 MS), Delay (−1 Immediate, +1 Delay), and Health Optimism (standardised) on our variables of face mask response efficacy and intention to wear a protective face mask.

**Figure 2:**
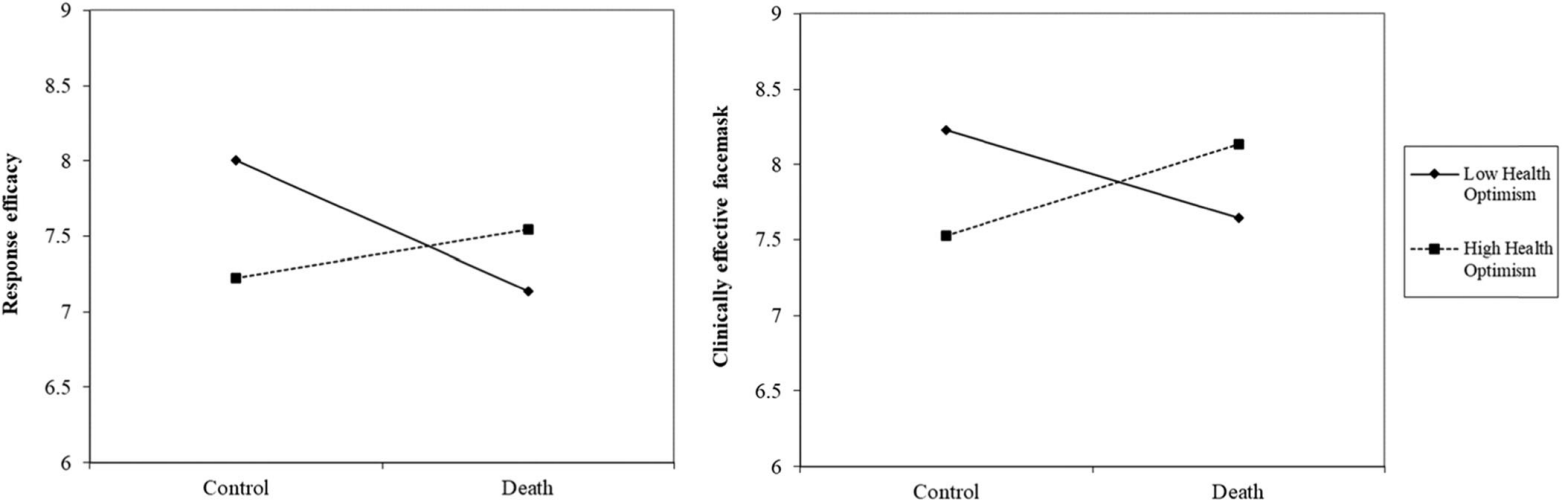
MS X Health Optimism on response efficacy of face masks and preference for a clinically effective face mask in the immediate condition plotted at ±1 *SD* levels of health optimism.

Concerning face mask response efficacy, there was a marginal MS X Delay interaction, *β* = .16, *t* (496) = 1.73, *p* = .084, 95% CI [−0.02, 0.34], that was qualified by the predicted three‐way interaction, *β* = −.20, *t* (496) = 2.14, *p* = .033, 95% CI [−0.38, −0.02]. Examining the MS X Health Optimism interaction at each level of the delay showed that the interaction was significant in the immediate condition, *β* = .33, *p* = .013, but not in the delay condition, *β* = −.06, *p* = .629 (see Figure 2).

Simple slopes analyses showed that at low levels (−1 *SD*) of health optimism, there was a significant effect of MS decreasing perceived response efficacy of face masks but only when measuring immediately, *β* = −.47, *t* (496) = 2.48, *p* = .014, 95% CI [−0.84, −0.10]. MS increased the perceived response efficacy of face masks for those high in health optimism when measuring immediately albeit this did not reach statistical significance, *β* = .19, *t* (496) = 1.06, *p* = .289, 95% CI [−0.16, 0.55]. Examining the effect of the delay yielded only an effect in those with low health optimism in the MS condition whereby the perceived response efficacy of face masks was decreased when measured immediately, *β* = .45, *t* (496) = 2.43, *p* = .015, 95% CI [0.09, 0.81].

Concerning intention to choose a clinically effective face mask, there was a significant MS X Delay interaction, *β* = .18, *t* (496) = 2.77, *p* = .006, 95% CI [0.05, 0.32], qualified by a marginal the three‐way interaction, *β* = −.11, *t* (496) = 1.70, *p* = .091, 95% CI [−0.24, 0.02]. Again, examining the two‐way interactions between MS X Health Optimism showed that the effect was only significant when measured immediately, *β* = .30, *p* = .002, but not when measured after a delay, *β* = .07, *p* = .440 (see Figure 2).

Simple slopes analyses showed that when measured immediately, MS decreased intention to wear a clinically effective face mask for those with low health optimism, *β* = −.29, *t* (496) = 2.14, *p* = .033, 95% CI [−0.56, −0.02], but MS increased intention for those with high health optimism, *β* = .30, *t* (496) = 2.29, *p* = .022, 95% CI [0.04, 0.56]. We also examined the effect of the delay, but this yielded no reliable effects, *p*'s > .15.

We next examined whether response efficacy mediated the effect of MS on decisions to wear a protective face mask using Model 12 in PROCESS (Hayes, 2018) using 10,000 bootstrapped samples. The three‐way interaction effect on intention to wear a protective face mask was significantly mediated by response efficacy, Index = −0.08, 95% CI [−0.17, −0.00]. Response efficacy significantly mediated the effect of MS on intention to choose a clinically effective face mask for those in low health optimism when measured immediately, *b* = −0.19, 95% CI [−0.37, −0.04]. No other effects were significant as the confidence interval included zero.

### 9.2 Appearance contingent self‐worth

**Figure 3:**
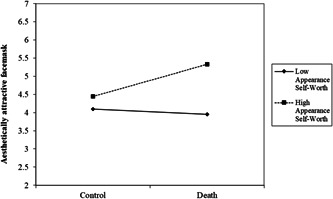
MS X appearance self‐worth on preference for aesthetically attractive face masks in the delay condition plotted at ±1 *SD* levels of appearance self‐worth.

We next examined our hypothesis that MS would increase the intention to wear a visually attractive face mask after a delay for those high in appearance contingent self‐worth. Running this analysis showed that there was a basic effect of increasing appearance contingent self‐worth preferring a visually attractive face mask, *β* = .46, *t* (496) = 5.27, *p* < .001, 95% CI [0.29, 0.63]. The only other effect close to significance was the three‐way interaction, *β* = .15, *t* (496) = 1.66, *p* = .097, 95% CI [−0.03, 0.32]. Examining the MS X Appearance Contingency interaction by each level of the delay showed that there was no effect immediately, *β* = −.04, *p* = .779, but there was a significant interaction in the delay condition, *β* = .26, *p* = .035. Figure 3 denotes this interaction.

Simple slopes analyses showed that after a delay for those high in appearance contingency, MS increased preference for a visually appealing face mask, *β* = .44, *t* (496) = 2.64, *p* = .009, 95% CI [0.11, 0.77]. No other effects were significant, *p*'s > .50. We also examined whether response efficacy mediated our delayed effects on a visually appealing face mask. As expected, this was not significant, *b* = 0.00, 95% CI [−0.04, 0.03].

### 9.3 Affect

We next examined whether our manipulations influenced levels of affect taking into account the role of health optimism. Our particular interest was on the fear subscale. The three‐way interaction was not significant, *β* = −.04, *t* (496) = 1.30, *p* = .194, 95% CI [−0.10, 0.02]. The other subscales were also not significant (*p*'s > 0.05).

Lambert and colleagues (2014) suggested that the fear subscale of the PANAS‐X is psychometrically questionable because it includes items that tap more into anxiety (i.e., jittery, nervous) than fear. Thus, we computed a different subscale that utilized the three items of the PANAS‐X (scared, frightened, afraid) in line with Lambert et al.'s (2014) suggestions and identical to some of the items of our fear composite in Study 1. The three‐way interaction on fear was now significant, *β* = −.08, *t* (496) = 2.37, *p* = .018, 95% CI [−0.15, −0.01]. Probing this interaction showed that the MS X Delay effect was only significant in those with low health optimism, *β* = .12, *p* = .015. Simple slopes analyses showed that MS increased fear for those with low optimism immediately, *β* = .22, *t* (496) = 3.46, *p* = .001, 95% CI [0.10, 0.35]. No other effects were significant, *p*'s > .25.

## 10 STUDY 2 DISCUSSION

The findings further supported the moderating role of health optimism in response to MS effects on COVID‐19 health behaviors. MS decreased the perceived response efficacy of face masks for those with low health optimism when measured immediately. Those high in health optimism tended to increase their perception of the response efficacy of face masks albeit not significantly. Additionally, MS marginally affected the intention to select a clinically effective face mask although this depended on levels of health optimism. Those low in health optimism tended to decrease their likelihood of picking a protective mask. Those high in health optimism tended to increase their likelihood of picking a clinically effective face mask. Additionally, the effect of low health optimism decreasing the likelihood of selecting a protective mask was mediated by decreases in response efficacy. This indirect effect might clarify the marginal nature of the interaction as there does not need to be a direct effect for an indirect effect to occur (Hayes, 2009). Further, we suspect that our lack of support for increases in perceived response efficacy, and the mediating role of perceived response efficacy, among those in health optimism are due to ceiling effects as responses were generally quite high on the scale.

Regarding distal defenses, our findings suggested that when thoughts of death were no longer conscious, MS increased preferences for an aesthetically appealing face mask, however, such an effect only reached conventional significance when examining the critical slope pertaining to our hypothesis. Consistent with our prediction, MS increased preferences for an aesthetically appealing face masks only when people felt that their appearance was an important source of their self‐worth. This effect was not mediated by response efficacy, and more generally MS did not increase interest in a clinically effective face mask after a delay. This further supports the idea that distal defenses are driven by concerns of self‐worth not health.

Our findings also partially corroborated the findings of Study 1 regarding fear. When utilising a specific composite of fear, we found that MS increased levels of fear immediately for those with low health optimism. However, we did not find that MS decreased levels of fear after a delay as in Study 1. One possibility is that this latter effect was spurious. Nonetheless, the immediate increase in fear after MS for those with low health optimism was confirmed in two large samples thus increasing confidence that it is a genuine effect.

## 11 GENERAL DISCUSSION

Since March 2020 when the pandemic officially began, people worldwide have been bombarded with various messaging and information about the COVID‐19 disease that has explicitly and implicitly increased death thoughts. For example, the number of death themes present in google searches, as well as social media, blog, and internet forum posts substantially increased after the pandemic started in comparison to before (Evers et al., 2021). This increase in death thoughts highlights the importance of the TMHM as a lens to understanding health behavior in the pandemic.

Across two studies we provided support for some of the ways that the awareness of death may influence health‐relevant constructs in relation to the COVID‐19 pandemic. The present findings showed that health optimism moderates proximal defenses to MS by influencing appraisals regarding one's perceived vulnerability to the COVID‐19 disease (Study 1), response efficacy of face masks (Study 2), and decisions to wear a face mask based on their clinical effectiveness (Study 2). The present research also demonstrated that COVID‐19 health preventative actions can also act as a distal response to MS when it appropriately taps into people's concerns over self‐worth. More specifically, those high in appearance contingencies of self‐worth showed increased interest in wearing aesthetically appealing face masks as a distal response to MS (Study 2). These findings can help inform ways in which we can combat the COVID‐19 pandemic.

First, our findings demonstrate how mortality‐based threats can produce proximal outcomes that are both health facilitating and health defeating. In particular, whether MS produces health facilitating or defeating outcomes depends on one's level of health optimism that can provide a sense of fortitude to manage health‐related threats. Our findings are therefore consistent with the proposition that optimism is linked with better health outcomes because it increases problem‐focused coping (e.g., Scheier & Carver, 1993). For example, those with low health optimism displayed an increased sense of vulnerability and fear, as well as a decreased perceived ability to cope with the COVID‐19 disease after MS. This experience of high threat and low perceived coping can produce outcomes that increase engagement with maladaptive behaviors (Rogers & Prentice‐Dunn, 1997). Such behavior is counter‐intuitive, but high vulnerability can engender fatalistic health beliefs (e.g., Valera et al., 2017), which might reflect how low health optimists engage in avoidance‐based coping strategies (Nes & Segerstrom, 2006). In the context of the pandemic, this might produce fatalistic outcomes such as not following advice regarding social distancing, isolation, handwashing, and face mask use. Indeed, our findings might correspond with research that shows that associating COVID‐19 with death is related to decreased intention to engage in health preventive action (e.g., handwashing, social distancing; Jimenez et al., 2020).

Our findings however suggest that such outcomes might be attenuated by ensuring messages are clear regarding how specific health behaviors, such as face masks, are highly effective. This corresponds with fear appeal research that suggests that efficacy statements increase the persuasiveness of the message (Tannenbaum et al., 2015; Witte & Allen, 2000). Our research however might also suggest when these fear appeals have the capacity to backfire. Specifically, unless effectiveness of health behaviors is emphasised, those who lack optimism might be particularly vulnerable to engaging in fatalistic tendencies after fear appeals, especially if such appeals arouse conscious death thoughts. Future research could examine the role of optimism in response to fear appeals further.

Our findings also noted that MS may immediately increase feelings of fear, which corresponds with past research (Lambert et al., 2014). Our findings add to this by suggesting that increases in fear are limited to specific individuals such as those who lack optimism that helps maintain confidence in the face of health threats. This finding might shed light on one reason why past research has generally struggled to identify that MS increases fear (although see Lambert et al., 2014 for other reasons), because it has generally not examined potential moderators to this effect.

While we did not predict an effect for fear, our finding could be considered consistent with our general assertions. High threat appraisal and low coping appraisal, as evinced by those with low health optimism across Studies 1 and 2, has been shown to elevate fear as a response to health threats (McGinty et al., 2012). According to the Extended Parallel Process Model (Witte, 1992), this increase in fear may also play a key role in producing maladaptive outcomes. However, because affect and our dependent measures were never measured at the same timepoint (i.e., participants either had their proximal fear and distal health behaviors measured or vice versa), we were not able to statistically examine the relationship between fear and our dependent variables, which limits our ability to draw firm conclusions about the role fear might play in our processes. While fear does not seem to drive distal defenses (Lambert et al., 2014), it is possible that it might drive proximal defenses (Juhl & Routledge, 2016). One possibility is that increases in fear prevents people from engaging in health preventative action. Alternatively, a sense of fear might stem from the fact that some people feel highly vulnerable to mortality threats and are ill‐equipped to combat them (i.e., MS → health behaviour → fear). Stated differently, when people feel they have the fortitude to manage health threats, this prevents increases in fear that might otherwise result from MS. Future research should examine these possibilities.

Regarding distal defenses, our findings suggest that considering ways in which health behaviors can be framed as a source of self‐worth or as being culturally valued may lead to better adherence to these health intentions long term. Unlike proximal defenses that are short‐lived, distal defenses have an enduring effect on people's behavior (see Morris et al., 2018), which can lead to more sustained engagement in COVID‐19 health preventative actions. For example, as evinced by our research, face mask use can be encouraged by making them visually appealing or as a way that people might people to assert their identity and/or ideological beliefs through logos, slogans, and brandings. Similarly, other health actions can be encouraged by highlighting the cultural value of these behaviors or increasing a sense of collectivism (Courtney et al., 2020; Courtney et al., 2021). For example, in the United Kingdom, messaging has often emphasised the role that people can have in combatting the pandemic (e.g., “play your part”). According to the TMHM, such discourse is likely to be effective because it implies that people can contribute to something greater than their mortal selves. It also likely taps into shared social identities and the potential they hold to encourage individuals to conform to perceived group norms (Castano & Dechesne, 2005).

To our knowledge, the present studies provide the most highly powered test of the TMHM claims so far. Despite this strength, our work does include some limitations. First, some of the findings in this paper were marginal which might question the reliability of those specific findings. This underscores the broader need for more terror management work that conceptually replicates findings using highly powered samples. Second, our studies relied on self‐report measures which are susceptible to socially desirable responding, especially given the nature of the research. This might explain why some of our findings may not have reached conventional thresholds of significance due to ceiling effects in our measures. Second, we are limited in making firm causal conclusions about the influences of vulnerability and response efficacy on health behaviors. For example, it is possible that the decrease in vulnerability we observed in Study 1 is because high health optimists base their sense of vulnerability on the fact that they intend to engage in health preventative actions. Based on the current data, we cannot discount this possibility.

Finally, one might question whether our effects are the result of terror management strategies or might be explained by increasing uncertainty (Van den Bos, 2009) or threats to meaning (Heine et al., 2006). Indeed, the pandemic has, for some, threatened one's sense of meaning (Todorova et al., 2021) and elevated feelings of uncertainty (Bakioğlu et al., 2021). These theories may therefore provide alternative ways to interpret our findings, however, equally feelings of meaning and certainty constitute important ways in which people manage feelings of death anxiety (Pyszczynski et al., 2015). Of course, these theories may not strictly be in a zero‐sum competition, and instead may illuminate on a set of unique, yet interrelated, concerns. For example, in the context of the pandemic, perhaps mortality‐based thoughts may heighten desires to affirm feelings of self‐worth, yet the concurrent threats to meaning and certainty that the pandemic provides might make it difficult for people to engage in effective terror management strategies. Future research could seek to examine the possible interface between these theories, though such issues went beyond the purview of our investigation.

Despite the worldwide vaccine rollout, we are a long way from defeating the COVID‐19 disease. New variants of the disease appear to be more resistant to the current vaccines (Fowlkes et al., 2021). This places emphasis on the need for continued engagement with health preventative actions such as using face masks if we are to continue to limit the spread of the disease, and ultimately save lives. Our findings may also offer ways to help public health officials consider how to encourage uptake of COVID‐19 vaccines, which remain our main hope of coming out of this crisis. For example, the knowledge that vaccines are highly effective in protecting from severe COVID‐19 disease may encourage vaccine use as a way of managing mortality‐based threats. Of course, the tendency to deny one's vulnerability to the COVID‐19 disease as a way of managing conscious death thoughts may reduce one's perceived need to take the vaccine. While messaging should ensure that these actions are seen as effective in reducing transmission and severe illness, it is also important that messaging considers how tapping into issues of self‐worth may motivate health preventative action. Ultimately, feeling like one can contribute to something greater than one's mortal self might be a better way to achieve sustained collective health action.

## CONFLICT OF INTEREST

The author declares no conflict of interest.

## Supporting information

Supporting information.

## ENDNOTES

1 Although some models of health behaviors imply that low threat appraisals result in no further evaluative response regarding coping appraisal (e.g., Witte, 1992), our contention here is that the reduced sense of vulnerability in response to MS reduces health threats to a level that are manageable, rather than eliminating the threat per se that would entail no need for a coping appraisal.

2 The online and MTurk versions of the survey were nearly identical aside from the following changes. First, we included several extra attention checks throughout the MTurk survey due to concerns over data quality. Second, because MTurkers work incredibly fast, we included a third delay task (the morningness‐eveningness questionnaire) to ensure a sufficient delay when measuring distal defenses.

3 We also had an auxiliary aim of examining whether MS would influence COVID‐19 conspiratorial beliefs. Therefore, some additional measures at the beginning of the study related to this aim (e.g., trust), and our dependent measures also included COVID‐19 conspiracy beliefs. Analyses of these scales produced no reliable effects. As they are unrelated to the current research aims we do not discuss them further. As our threat appraisal scales were always answered before the conspiracy questions, we are confident that these measures did not confound our findings.

4 We also measured vaccine intention because we were interested whether our manipulations had an indirect effect on intention. Our findings supported this (see Supplementary Analyses). However, due to administrative issues in data collection, most participants reported having already taken the vaccine. Although, intention may reflect willingness to take second doses or booster vaccines, conceptually it is difficult to feel confident in this interpretation as the items did not specifically mention these, thus we do not report it here.

5 We also reran the analyses controlling for vaccine status (dummy coded). Controlling for this did not change the findings, though the three‐way interaction was now only marginal, *β* = .16, *t* (434) = 1.80, *p* = .072, 95% CI [−0.01, 0.33]. The immediate effect of MS at high levels of health optimism was still significant, *β* = −.37, *t* (434) = 2.20, *p* = .028, 95% CI [−0.70, −0.04], and the effect at low levels of health optimism was still not significant, *β* = .21, *t* (434) = 1.14, *p* = .255, 95% CI [−0.15, 0.57].

6 Two participants did not answer the specific items pertaining to fear so were excluded from this analysis.

7 We also measured the perceived value of wearing a face mask because we had an auxiliary hypothesis that there may be distal effects of wearing a protective face mask because the behavior is seen as culturally valued. However, the effects for this measure were not reliable, possibly because of ceiling effects in the measure (*M* = 7.9).
