# Supplementary material for: The proximal and distal effects of mortality salience on COVID‐19‐related health perceptions and intentions
Source: J Appl Soc Psychol. 2022 Jul 5:10.1111/jasp.12903. Online ahead of print. doi: 10.1111/jasp.12903 (PMC9349686; doi:10.1111/jasp.12903)
Supplement: Supplementary file 1 — Supporting information. [file JASP-9999-0-s001.docx]

**Supplementary Analyses**

In Study 1, we had intended to examine how our manipulations might influence vaccine intention (measured using 3 items). However, due to administrative issues, we were delayed in conducting the study, and the majority of the data was collected well into the vaccine rollout. This meant that a vast majority of participants had already taken, or opted not to take, a COVID-19 vaccine. While it is possible that the findings could still reflect intention for future COVID-19 vaccinations (e.g., second dose, booster jabs), given our measure did not specifically mention these possibilities it is unclear whether participants necessarily answered with these possibilities in mind. Thus, we believe that the findings presented in the Supplementary Analyses should be taken with caution.

***Vaccine intention***

We first examined if there were any direct effects on our vaccine intention measure. There was an effect of health optimism decreasing vaccine intention, β = -.63, *t* (436) = 4.56, *p* <.001, 95% CI [-.90, -.36]. The three-way interaction was not statistically significant, β = .22, *t* (436) = 1.62, *p* = .106, 95% CI [-.05, .49]. Noting the non-significant interaction, we continued on to examine the simple slopes. MS decreased vaccine intention for those high in health optimism when measured immediately, β = -.58, *t* (436) = 2.23, *p* =.026, 95% CI [-1.09, -.07]. The other slopes were not significant, *p*’s *> .*35. We also examined the effect of the delay, and the only effect approaching significance was those high in health optimism in the MS condition decreasing their vaccine intention when measured immediately, β = .49, *t* (436) = 1,73, *p* = .084, 95% CI [-.07, 1.05]. All other effects were not significant, *p*>.20.

As our hypothesis pertained more to the indirect, rather than direct, effects of MS on vaccine intention we sought to examine any indirect effects using Model 12 in PROCESS (Hayes, 2018), which can test for a three-way interaction effect on vaccine intention through a mediating variable (perceived vulnerability). The 95% CIs for the indirect effect were estimated via bootstrapping analyses using 10,000 samples. The overall effect of the three-way interaction on vaccine intention was significantly mediated by perceived vulnerability, Index = .08, 95%CI [.01, .16]. MS indirectly decreased vaccine intention but only among those high in health optimism when measured immediately, b = -.17, 95%CI [-.35, -.02]. There was also an indirect effect of the delay in the MS condition, whereby vaccine intention increased when measured immediately for those with low health optimism, b = -.16, 95%CI [-.32, -.02].
